# Water storage paradox of reservoir expansion and evaporative losses in the MENA region

**DOI:** 10.1038/s41598-025-21859-w

**Published:** 2025-10-01

**Authors:** Milad Aminzadeh, Sankeerth Narayanaswamy, Hannes Nevermann, Matteo Zampieri, Ibrahim Hoteit, Paolo D’Odorico, Amir AghaKouchak, Kaveh Madani, Nima Shokri

**Affiliations:** 1 Institute of Geo-Hydroinformatics, Hamburg University of Technology, Hamburg, Germany; 2 United Nations University Hub on Engineering to Face Climate Change at the Hamburg University of Technology, United Nations University Institute for Water, Environment and Health (UNU-INWEH), Hamburg, Germany; 3 Physical Sciences and Engineering Division, King Abdullah University of Science and Technology, Thuwal, Saudi Arabia; 4 Climate Change Center (CCC), National Center for Meteorology (NCM), Jeddah, Saudi Arabia; 5 Department of Environmental Science, Policy, and Management, University of California, Berkeley, CA USA; 6 Department of Civil and Environmental Engineering, University of California, Irvine, CA USA; 7 United Nations University Institute for Water, Environment and Health (UNU-INWEH), Richmond Hill, ON Canada

**Keywords:** Climate sciences, Environmental sciences, Hydrology, Water resources

## Abstract

Prolonged droughts and population growth have increased the demand for efficient water storage globally. Small agricultural reservoirs support local water demands, but high evaporation rates particularly in dry regions undermine their storage effectiveness. Integrating fine-resolution Sentinel-2 imagery and physical modeling, we created an annual dataset of small agricultural reservoirs (< 0.1 km^2^) in the Middle East and North Africa (MENA) and quantified their associated evaporative losses from 2016 to 2023. We identified over 133,700 reservoirs, peaking in 2020, providing a combined surface area of 1,408 km^2^. The largest cumulative areas are located in Türkiye (309 km^2^), Pakistan (234 km^2^), Iran (168 km^2^), Iraq (108 km^2^), and Egypt (64 km^2^). Small agricultural reservoirs offer a storage capacity of 1,243 million cubic meters, accounting for up to 16% of irrigation and livestock water use in most MENA countries. Annual evaporative losses from these reservoirs may potentially exceed 2,400 million cubic meters with hotspots of cumulative evaporation corresponding to regions with the highest reservoir surface area, including southern Pakistan, southwestern Iran, and southeastern Iraq. Our analysis suggests strong climatic and anthropogenic influences on the expansion of reservoirs and their storage efficiency emphasizing the need for mitigation strategies to improve agricultural water security in water-stressed regions.

## Introduction

Freshwater shortages have been intensified by the rising water demands of a rapidly growing population and shifts in precipitation and drought patterns^1,2^. It is estimated that approximately 4 billion people experience severe water scarcity for at least one month each year, while around 500 million are affected year-round^3^. Transboundary conflicts and political instability, such as those affecting the Nile Basin, may further exacerbate water insecurity^4–7^. This makes agricultural production potentially vulnerable to the availability of surface and groundwater resources (i.e., sizable lakes, aquifers, and reservoirs), particularly in arid and semi-arid regions with sparse riparian networks and limited natural lakes^8,9^.

On-farm water reservoirs are at the core of supporting local irrigation and livestock water demands in dry spells^10,11^. However, small agricultural reservoirs (often smaller than 0.1 km^2^) are commonly ignored in the global studies of artificial water reservoirs^12^ and in the surface water datasets such as HydroLAKES^9^ and GRanD^13^. Invoking statistical approaches (e.g., the Pareto model), the total number of artificial reservoirs and ponds between 0.001 and 0.1 km^2^ is estimated at approximately 4.29 million^14^. They require fewer resources for construction and implementation at the farm level compared to large-scale storage infrastructures such as dams^15–17^.

Agricultural activities account for over 80% of freshwater withdrawals in the Middle East and North Africa (MENA), with farming production heavily reliant on irrigation^18–20^. Despite the crucial role of agricultural reservoirs in enhancing food security and alleviating poverty, particularly in less-developed regions^17^, the impact of these small on-farm reservoirs on the management and allocation of dwindling freshwater resources remains largely unexplored. Similar to the impact of climate change and rising temperatures on intensifying evaporative water loss from surface reservoirs (e.g., lakes and dams)^21–23^, small water storages are increasingly vulnerable to evaporation losses in a warming world^11^. This makes accurate estimation of water evaporation from small water reservoirs in the MENA region crucial for evaluating reservoirs’ storage efficiency^24^. Evaporative loss of stored water in agricultural reservoirs poses serious risks to groundwater resources, as in many regions these reservoirs are primarily fed by groundwater^19,25^. Simply put, when evaporative losses from these reservoirs are not considered for water management, it not only impacts agriculture, crop production, and the local economy, but also leads to the irreversible depletion of this vital resource^26,27^. Given the ongoing changes in water systems due to climate variability and extreme events, and in some cases poor management, this often-overlooked factor, particularly in the absence of robust monitoring, could exacerbate regional and transboundary water disputes as demand continues to increase^28^.

We aim to address this knowledge gap by integrating fine-resolution satellite imagery with physics-based modeling to provide a systematic assessment of small agricultural reservoirs (< 0.1 km^2^) across the MENA region from 2016 to 2023, quantifying both their annual storage capacity and evaporative losses. By linking the extent of these water reservoirs to changes in freshwater availability and demands arising from climatic factors and agricultural activities, our study identifies hotspots of potential water loss and informs strategies for improved water management and transboundary cooperation in this water-stressed region.

## Results

### Spatio-temporal distribution of water reservoirs and their storage capacity

**Fig. 1:**
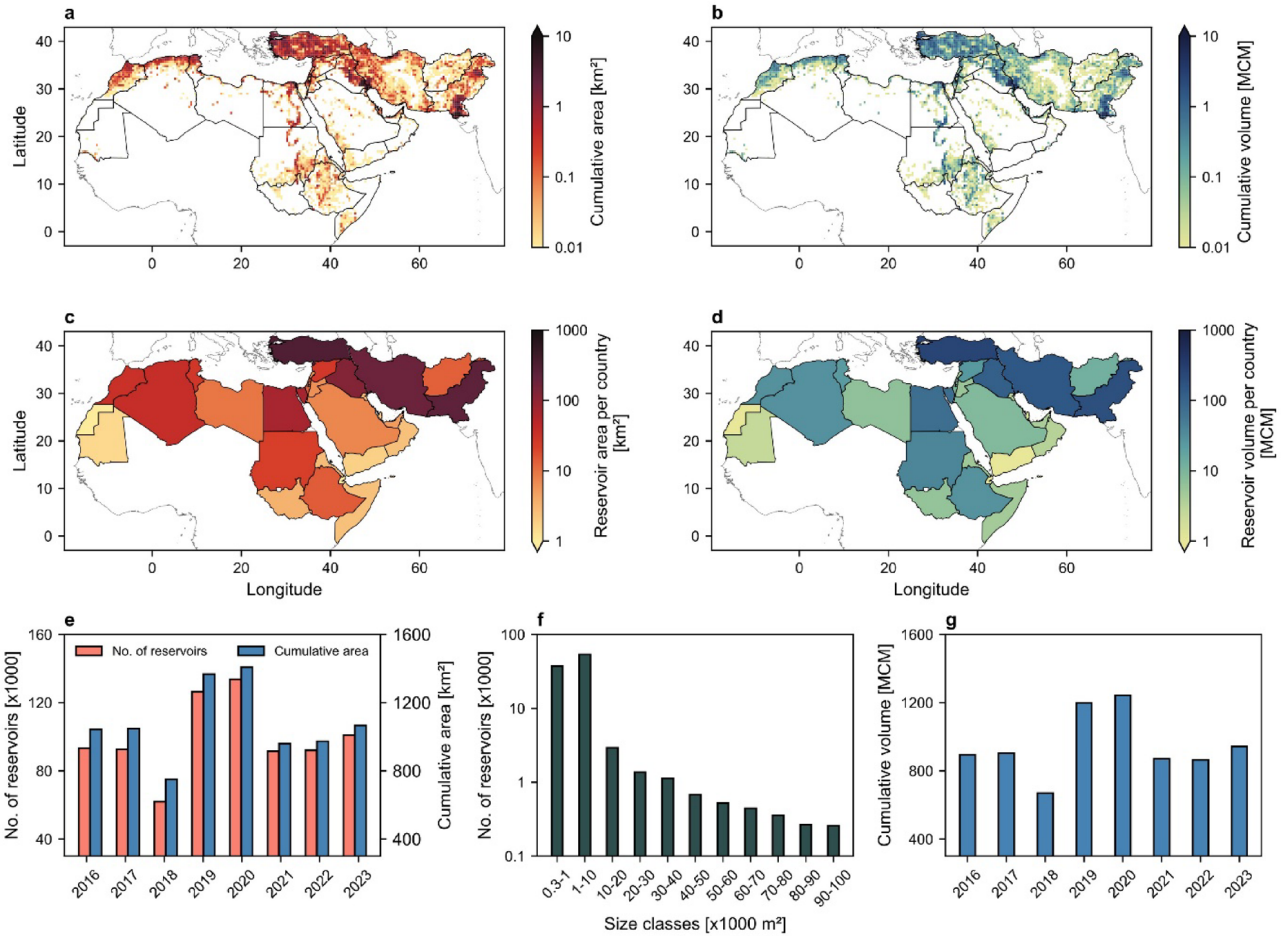
Spatial distribution and characteristics of small agricultural reservoirs in MENA. The mean cumulative surface area (**a**) and storage capacity (**b**) of small agricultural reservoirs in each 50 × 50 km^2^ grid cell in the study area (-18.42° to 78.60°E, -1.80° to 44.47°N) from 2016 to 2023. Total reservoir area per country (**c**) and total reservoir volume per country (**d**). Temporal variation of total number of reservoirs and their cumulative area (**e**); the size distribution of reservoirs in MENA with 11 size classes (**f**); and total storage capacity of reservoirs (**g**).

Highly resolved Sentinel-2 satellite images with 10 m resolution were used to identify the spatio-temporal distribution of water reservoirs smaller than 0.1 km^2^ in the MENA region from 2016 to 2023 (Fig. 1). The study area was partitioned into 50 × 50 km^2^ grids to determine the total number and cumulative area of small reservoirs within agricultural areas across the MENA region (see Methods for details). The storage capacity of the reservoirs in each grid cell was further estimated based on their size distribution, following the power-law relationship proposed by Mady et al.^10^. The total number of reservoirs in the agricultural area of MENA changed from approximately 93,300, covering an area of 1,044 km^2^ in 2016, to ~ 101,000 reservoirs with a total area of 1,067 km^2^ in 2023. The analysis indicated that the number of reservoirs peaked at around 133,700 in 2020, with a cumulative area of 1,408 km^2^. The highest storage capacity was also observed in 2020 at 1,243 million cubic meters (MCM), while the lowest was recorded in 2018 at 670 MCM. Yearly variations of cumulative surface area and storage capacity in the study area are provided in the Supplementary Information (Figures S1 and S2).

The results indicate that small reservoirs are primarily aggregated in southern Pakistan, Türkiye, southwestern Iran and southeastern Iraq with considerable agricultural activities (Fig. 1a and b). The highest number of reservoirs was observed in Pakistan with 20,900 reservoirs covering 234 km^2^ and total storage capacity of 182 MCM. Meanwhile, Türkiye had the largest cumulative reservoir area, totaling 309 km^2^, with a storage capacity of 258 MCM (Fig. 1c and d). The highest density of reservoir area relative to the cropland area is detected in Bahrain, UAE, Qatar, Western Sahara, and Kuwait with 0.194, 0.032, 0.025, 0.014, and 0.006 km^2^ reservoir area per square km of cropland, respectively. Extremely low precipitation in these countries, ranging from 50 to 110 mm/year^29^, underscores the critical need for water storage infrastructure to sustain agriculture.

### Evaporative losses from agricultural reservoirs

Although agricultural reservoirs and engineered impoundments are often lined with waterproof barriers to reduce seepage losses, the hot and dry climatic conditions in the MENA region significantly increase evaporative losses from reservoirs. We used a physically-based model^30^ to quantify evaporation from small water reservoirs (see Methods section). Due to the lack of detailed information on the depth of individual reservoirs, we relied on the analysis of Mady et al.^10^ suggesting that the effective water depth in agricultural reservoirs smaller than 0.1 km^2^ ranges between 2 and 4 m. The model thus allowed us to calculate evaporation loss from a typical reservoir with an average depth of 3 m in each grid cell subjected to local meteorological conditions including radiation, wind, air temperature, and humidity, extracted from the Modern-Era Retrospective analysis for Research and Applications, Version 2 (MERRA-2)^31,32^. Small agricultural reservoirs may remain empty during certain periods of the year, and the water level within individual reservoirs may dynamically change due to on-farm management practices. Therefore, we estimated evaporative losses under the simplifying assumption that reservoirs remain full throughout the year, providing an upper-bound estimate of potential water loss via evaporation.

**Fig. 2:**
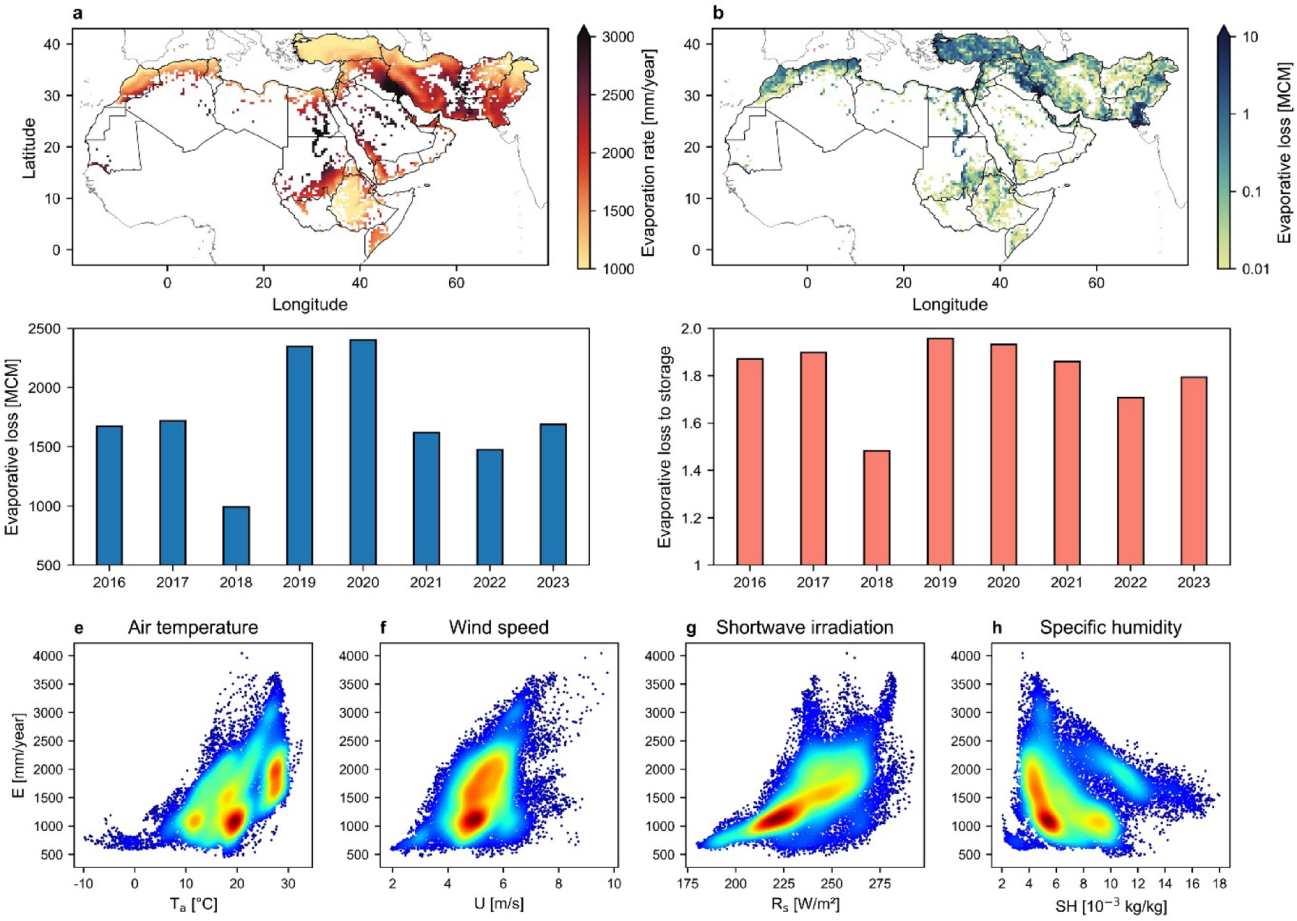
Dynamics of evaporative water loss from small agricultural reservoirs. (**a**) Model estimates of mean yearly potential evaporation rate from small reservoirs during the study period (2016–2023). (**b**) Mean values of annual cumulative evaporative water loss from water reservoirs in each grid cell (2016–2023). (**c**) Yearly variation of total evaporative loss from agricultural water reservoirs in the MENA region. (**d**) The ratio of annual evaporative loss to total water storage capacity of reservoirs. (**e**–**h**) Changes in evaporation rate from reservoirs in individual grid cells with variation of mean annual air temperature, wind speed, shortwave radiation, and specific humidity.

Figure 2a shows the spatial variation in the mean yearly evaporation rate (mm/year) from typical agricultural water reservoirs across grid cells during the study period (2016–2023). Model estimates of evaporative losses from agricultural reservoirs were primarily validated using direct measurements from small water storages (Figure S3). We used detailed evaporation observations from two small reservoirs: Cartagena, Spain (April 2007–March 2008), and Isfahan, Iran (April 2019–March 2020). Both reservoirs were lined with waterproof materials along the bottom and side walls and water level changes due to evaporation were monitored using pressure transducers. Comparison of measured and modeled monthly evaporation showed good agreement with an RMSE of 14.38 mm (see Supplementary Information for details). Yearly potential evaporation rate from agricultural reservoirs across the MENA region are provided in Figure S4. The results in Fig. 2a indicate exceptionally high atmospheric evaporative demand in eastern and southwestern Iran, southeastern Iraq, central regions of Saudi Arabia, and along the Nile River in Sudan and Egypt, where evaporation rates from shallow water reservoirs exceed 3,000 mm/year. These findings align with measured evaporative losses in regions such as southeastern Iran, which experiences high temperatures and extremely strong winds that can easily surpass 100 km/h during the summer months (the so-called 120-day winds in the Sistan region), thus causing intensified evaporative losses from water surfaces^6,7^.

Quantifying the extent of water reservoirs in each cell enables the calculation of cumulative evaporative losses for that cell. Figure 2b depicts spatial variation of mean yearly evaporative losses in the MENA region from 2016 to 2023 (see annual details in Figure S5). Hotspots of cumulative evaporative losses correspond to regions with a high surface area of reservoirs, as depicted in Fig. 1a (i.e., southern Pakistan, southwestern Iran, and southeastern Iraq). Variation in total evaporative losses from small agricultural reservoirs in MENA (Fig. 2c) reveals a mean annual evaporation of 1,740 MCM with peak losses that may exceed 2,400 MCM/year. We further investigated the evaporation rates for reservoir depths of 2 and 4 m (Figure S6). Shallower reservoirs typically experience higher rates of evaporation due to their lower thermal inertia and energy storage capacity which allows for quicker warming and increased evaporation rates in response to variation of solar radiation and ambient temperature in summer months^30^. Our results revealed slight variations in evaporation losses when comparing depths of 2 to 4 m, suggesting that while reservoir depth influences evaporation dynamics, other factors such as climatic conditions and reservoir surface area play a dominant role in determining overall losses via evaporation. This is evidenced by the general trend of total evaporative loss in Fig. 2c which follows the yearly variations of total surface area of the reservoirs depicted in Fig. 1e.

The annual total evaporative losses relative to the storage capacity of the reservoirs from 2016 to 2023, shown in Fig. 2d, indicate that the volume of water potentially lost through evaporation could be as much as double the storage capacity of the reservoirs. This highlights the paradox of storing valuable blue water in small reservoirs where high evaporation rates undermine storage efficiency; thus emphasizing the urgent need for targeted management practices such as evaporation suppression techniques and improved reservoir design^30,33–36^. Such measures could significantly enhance water conservation efforts and promote sustainable water management, particularly in arid regions facing severe water scarcity problems. As noted, reservoirs may remain empty during certain periods of the year; thus, the values in Fig. 2c represent an upper-bound estimate of potential evaporative losses. Likewise, the results in Fig. 2d assume each reservoir is filled only once per year, whereas in reality many are refilled multiple times meaning cumulative stored water can exceed the estimates shown in Fig. 1g. These results therefore highlight the vulnerability of small reservoirs to high evaporative demand, rather than implying complete depletion.

Our grid cell analysis of evaporation from agricultural reservoirs marks the key role of climatic factors in driving evaporative losses (Fig. 2e-h) showing that evaporation increases with rising air temperature, wind speed, and solar radiation, but decreases with higher humidity in the MENA region. Results indicate that mean annual air temperature, wind speed, and shortwave radiation in grid cells exceed 30 °C, 8 m/s, and 275 W/m^2^, respectively, while specific humidity can drop to as low as 0.002 kg/kg, marking the high atmospheric evaporative demand in the MENA region. Understanding the impact of these primary climatic factors is essential for identifying areas susceptible to high evaporative losses and implementing effective strategies in vulnerable regions.

By quantifying the spatial distribution of evaporative losses from reservoirs across the MENA region, we were further able to trace the fate of evaporated water using atmospheric moisture trajectory analysis (Figure S7). The results revealed that local recirculation of evaporated water from small agricultural reservoirs in MENA is minimal; instead, the majority is dispersed and transported to distant regions far from its source.

### Climatic and agricultural drivers of reservoir expansion in the MENA region

**Fig. 3:**
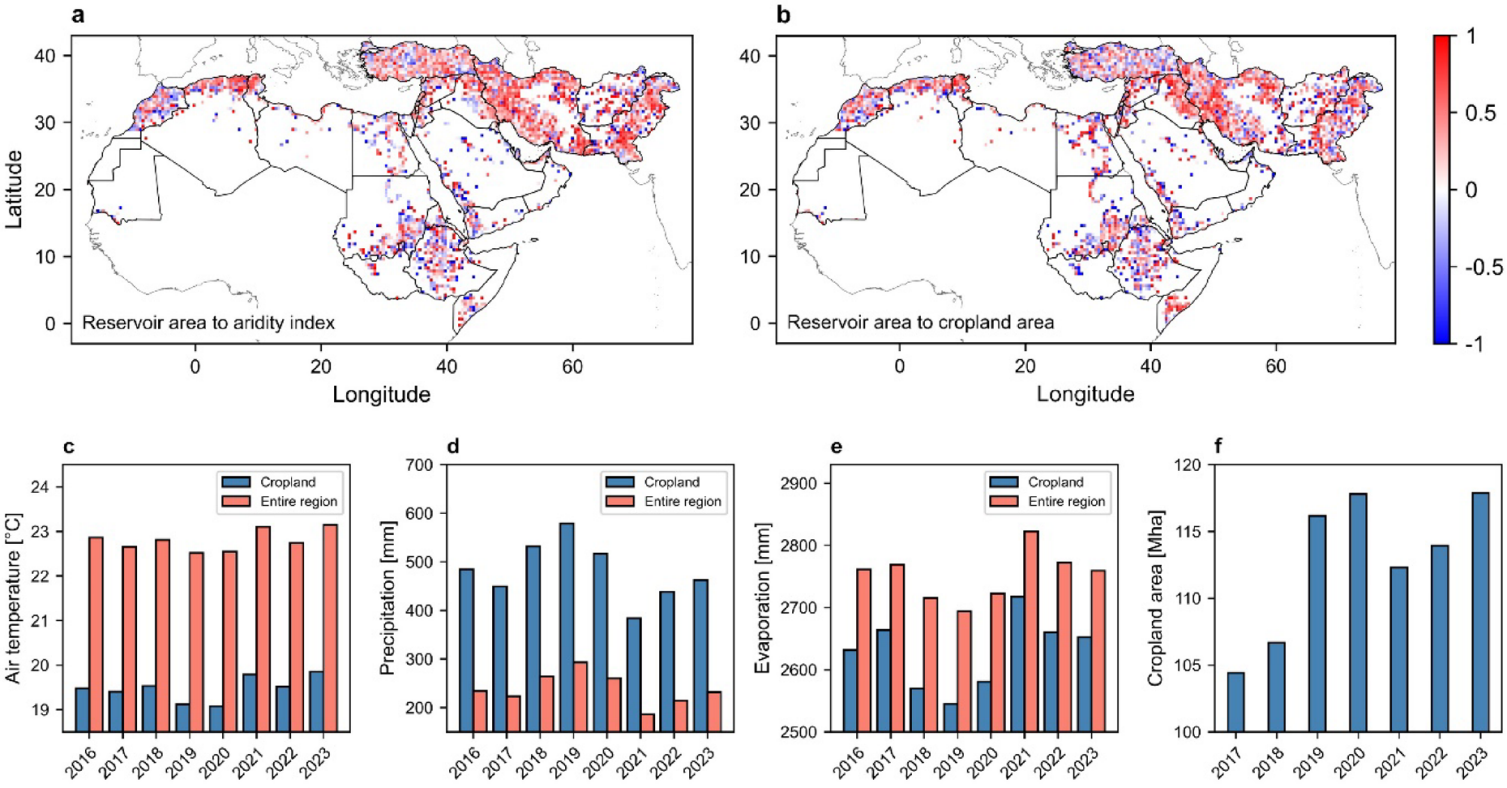
Correlations between the extent of water reservoirs and climatic and land use factors. Spatial variation of Pearson correlation coefficient between the cumulative surface area of water reservoirs and aridity index (**a**), and cropland area (**b**). Variation of mean air temperature (**c**), annual precipitation (**d**), annual evaporation (**e**), and cropland area (**f**) from 2016 to 2023 in MENA.

The extent of water-filled agricultural reservoirs, as depicted in Fig. 1, is likely linked to variations in freshwater availability and demand, influenced by climatic factors and agricultural activities. Understanding these interconnections requires investigating the influence of key climatic variables, particularly precipitation and temperature. Figure 3 illustrates grid cell analysis of the variation of Pearson correlation coefficients between cumulative reservoir area and aridity index (the ratio of precipitation to potential evapotranspiration), and cropland area for 2016–2023. The analysis shows positive correlations with aridity index suggesting that the expansion of water reservoirs is likely a response to climate-induced changes in water availability. While increased precipitation (which raises the aridity index) enhances water availability and storage in agricultural reservoirs, rising air temperatures increase evapotranspiration (lowering the aridity index) and reduces water availability for reservoir filling.

A detailed analysis of climatic trends in the MENA region during the study period reveals an increase in air temperature and evaporation rates, alongside a decline in precipitation (Fig. 3c–e). The rate of these changes is more pronounced in cropland areas compared to the entire region. For instance, while yearly precipitation across the entire MENA region has decreased at a rate of 3.9 mm/year during the study period, the decline of precipitation in croplands is more than double, at 8.5 mm/year. Comparing the temporal variations in cumulative reservoir area shown in Fig. 1e with the changes in precipitation patterns depicted in Fig. 3d demonstrates that the cumulative surface area of reservoirs responds to fluctuations in precipitation and, consequently, water availability (Figure S8).

Climatic factors alone cannot fully explain the expansion of small reservoirs; socioeconomic and political dynamics also play a critical role in shaping water management strategies across the MENA region. While our results provide useful insights, a longer study period and detailed local information on reservoir management would be required to more clearly disentangle climate-driven from management-driven influences and to explain the contrasting correlations observed among neighboring cells. Approaches such as time-series analysis, distributed lag models, or non-parametric correlations could better capture lagged and non-linear responses in reservoir-climate-agriculture interactions, particularly when investigating the interannual dynamics of reservoirs. The significant expansion of agricultural areas, with croplands increasing by 1.82 million hectares per year during the study period, suggests that factors such as population growth, policy interventions, and economic drivers likely contributed to the rising demand for irrigation and water storage. Accordingly, correlation with cropland area in our analysis should be considered as a proxy for agricultural expansion influencing reservoir development, rather than a direct measure of irrigation water demand.

In summary, while climatic factors (represented here by aridity index) provide valuable insights into regional water availability, their interplay with socioeconomic and geopolitical drivers over longer periods must be considered when assessing changes in water storage infrastructure of the MENA region. Construction of the Grand Ethiopian Renaissance Dam^4,5^ which has led to water disputes between Ethiopia, Egypt, and Sudan, and expanding water storage infrastructures in the Helmand basin shared by Afghanistan and Iran^6,7,37^ are prime examples of anthropogenic factors that may influence downstream water availability and, consequently, small reservoirs dynamics in MENA.

### Implications of small reservoirs for local water management

Integrating small on-farm reservoirs into broader water management strategies can enhance resilience against climate variability and improve food security in vulnerable regions. Agricultural reservoirs serve as a buffer for irrigation and livestock water demands, thereby stabilizing agricultural production during dry spells. They are particularly useful in supporting drip irrigation systems by providing a reliable and controlled water supply that ensures crop needs are met while minimizing water loss in precision agriculture^38^.

**Fig. 4:**
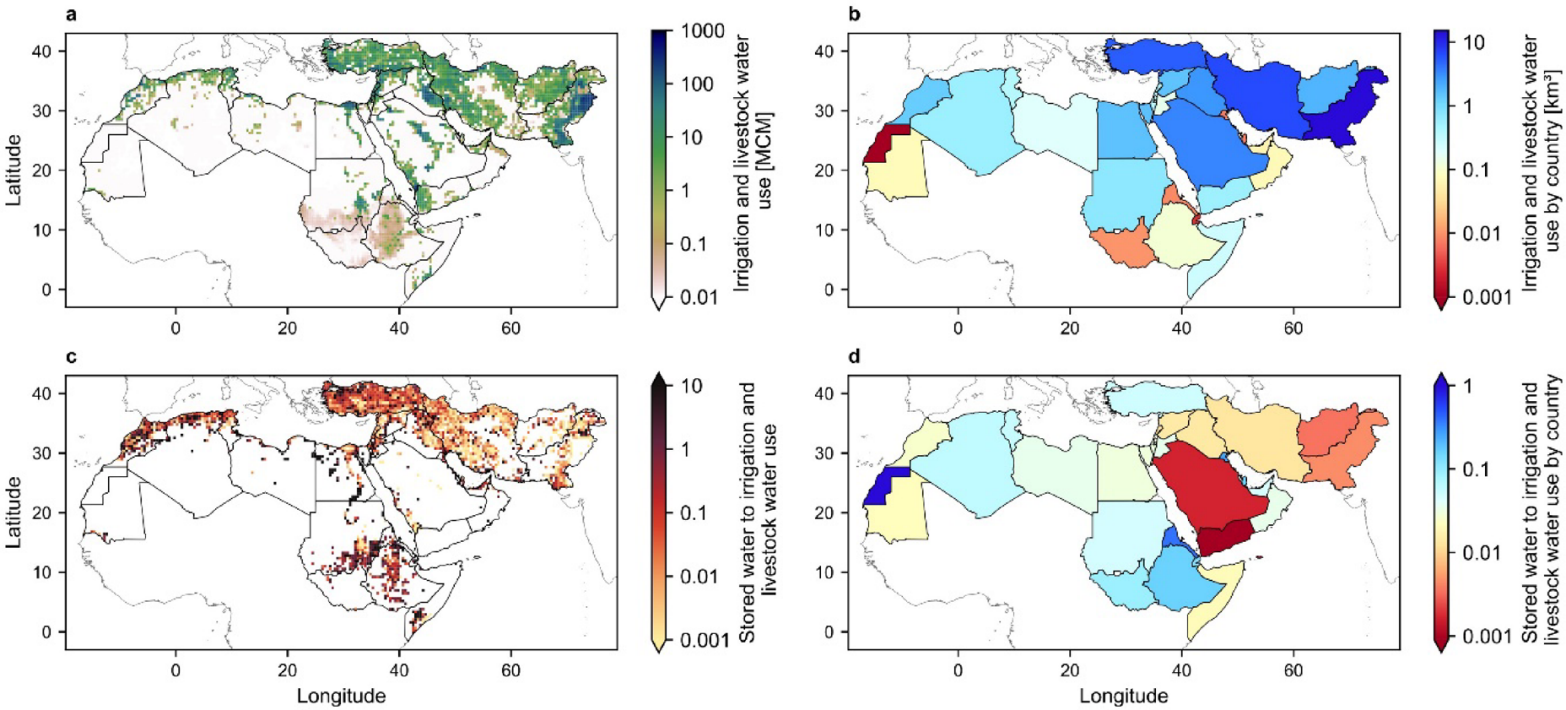
Irrigation and livestock water use in the MENA region. (**a**) Spatial distribution of irrigation and livestock water use in the MENA region. (**b**) Irrigation and livestock water use in each country. The color bar extends to 15.29 km^3^. (**c**) The ratio of cumulative stored water in each grid cell to the irrigation and livestock water use in that cell. (**d**) The ratio of stored water in small agricultural reservoirs to the total irrigation and livestock water use in each country.

Figure 4a shows grid cell analysis of the annual irrigation and livestock water use across the MENA region (Methods)^39,40^. Pakistan, Iran, Türkiye, Saudi Arabia, and Iraq have the highest irrigation and livestock water use with 15.29, 6.55, 5.19, 3.41, 2.92 km^3^, respectively (Fig. 4b), reflecting their significant dependence on irrigation to sustain crop production. These countries rely heavily on both surface and groundwater resources to meet their agricultural demands, which are further exacerbated by arid climates and the growing water demands. While green water (i.e., root zone soil moisture from rainfall inputs) plays a critical role in meeting crop needs, small reservoirs in some grid cells significantly contribute to water supply (Fig. 4c). Note that this first-order estimation tacitly assumes these reservoirs are filled only once per year. In practice, however, reservoirs could be refilled multiple times annually suggesting that the values shown in Fig. 4c likely represent a conservative estimate of their contribution in supplying local demands. Small agricultural reservoirs account for up to 16% of irrigation and livestock water use in most MENA countries (Fig. 4d). Western Sahara (264%), Bahrain (55%), Eritrea (39%), Kuwait (25%), and Ethiopia (16%) have the highest reservoir storage capacity relative to their irrigation and livestock water use.

Agricultural reservoirs capture and store runoff during rainfall thus reducing flood risks and preventing soil erosion which are both essential for maintaining productive farmlands. However, effective management of these reservoirs requires careful planning based on local climate patterns, hydrology, and land use to optimize their impact on water conservation and agricultural sustainability. In particular, significant evaporative losses from open water surfaces in MENA which might exceed 3,000 mm/year must be addressed to improve the storage efficiency of agricultural reservoirs in water-scarce areas through new innovative approaches^30,35,36^.

## Discussion

### Global abundance and environmental impacts of agricultural reservoirs

Our study provides the first regional inventory of small agricultural reservoirs (< 0.1 km^2^) across the MENA region for 2016–2023. The number of reservoirs peaked at ~ 133,700 in 2020, with hotspots concentrated in southern Pakistan, Türkiye, southwestern Iran, and southeastern Iraq. Together, these reservoirs offer an estimated storage capacity of ~ 1,243 MCM, but are subject to extreme evaporative losses (exceeding 3000 mm/yr). Our findings resonate with global studies showing similar challenges in other semi-arid regions. In Australia, for instance, small on-farm reservoirs have expanded at ~ 1.2% per year since 2000 ^41^, with evaporation accounting for a substantial fraction of stored water^42^. Comparable issues have been reported in South America and Africa, where small reservoirs play a central role in agricultural water supply but suffer from high evaporative losses^43,44^. These underscore both the urgency of incorporating on-farm reservoirs and their evaporative losses into regional freshwater assessments and the need to prioritize management interventions that account for efficiency, cost, and feasibility.

Beyond agriculture, the rapid growth of small reservoirs influences a wide range of environmental processes. They provide habitat for water birds, breeding grounds for amphibians, and improved conditions for aquatic macroinvertebrates, but agricultural reservoirs have also been identified as notable sources of greenhouse gas emissions, with some of the highest emission rates per unit area among freshwater systems^41,45,46^. They may further intensify water conflicts and exacerbate drought conditions in downstream areas (e.g., reservoirs conflicts along the Mekong river)^47^. This dual role, offering local benefits while generating wider hydrological and environmental trade-offs, illustrates the complex management challenge posed by small reservoirs.

Despite their significance, reliable data on the spatial distribution, density, and temporal dynamics of small reservoirs remain limited, particularly in developing and water-stressed regions. This data gap constrains evidence-based policy and sustainable water management. By producing the first annual, fine-resolution inventory of small agricultural reservoirs and their evaporative losses across the MENA region, our study fills a critical knowledge gap and provides a benchmark for assessing similar challenges in other semi-arid regions worldwide.

### Limitations and future perspectives

This study provides a framework to estimate spatial and temporal distribution of small agricultural reservoirs and their evaporative losses thereby contributing to more accurate water accounting, particularly in water-stressed regions. However, this approach has certain limitations. Our water detection algorithm primarily depends on Sentinel-2 multispectral imagery, which is highly sensitive to cloudiness. This limitation restricts the monitoring of reservoir dynamics, especially during rainy seasons with frequent cloud cover. To overcome this challenge, future research could integrate Sentinel-1 imagery, which utilizes Synthetic Aperture Radar (SAR) to capture microwave signals that are less influenced by weather conditions^48^. By combining optical and radar-based imagery, the accuracy and consistency of water reservoir detection could be significantly improved, especially in regions with frequent cloud cover^49^. Given the broad spatial extent of the study region, a threshold-based spectral index (the Normalized Difference Water Index – see Methods) was adopted as a practical and computationally efficient method for water detection^50^. While variability in surface and environmental conditions may affect our detection accuracy, future work could benefit from integrating multiple spectral water indices combined with dynamic thresholding techniques to improve the robustness and adaptability of surface water detection^50,51^.

Our storage capacity estimates rely on a generalized power-law relationship^10^ which may introduce uncertainty when applied to individual reservoirs. The lack of detailed local bathymetric surveys in most MENA countries prevents direct validation against official records. Future studies should incorporate locally calibrated area-volume functions, where available, to improve the accuracy of volume estimation. Model estimates of evaporative losses from water reservoirs are influenced by the spatial and temporal resolution of meteorological inputs which are currently obtained from MERRA-2. While the hourly temporal resolution of MERRA-2 facilitates the calculation of dynamic evaporation changes, the introduction of high spatial resolution atmospheric data, such as the 1 km-scale climate information expected from the Destination Earth project^52,53^, will allow for more precise evaporation estimates with much improved spatial resolution.

Finally, our estimates of water use in the agricultural sector are based on the global irrigation dataset provided by Zhang et al.^54^. This dataset integrates various satellite-based soil moisture and precipitation products and develops a physically-based framework for estimating global irrigation water use with multiple satellite observations. While this enables consistent regional comparisons, it does not capture local irrigation practices (e.g., pressurized irrigation) and their impacts on agricultural water use, which may cause discrepancies with local or national estimates. Access to accurate official data across the MENA region, along with more detailed irrigation datasets, would allow a more realistic assessment of the contribution of small on-farm reservoirs to agricultural water use and food production.

## Methods

### Identification of small agricultural reservoirs and estimating their storage capacity

We used Sentinel-2 satellite images (launched in 2015) with 10 m resolution to identify small water reservoirs (< 0.1 km^2^) in agricultural areas of the MENA region from 2016 to 2023. The study region was divided into grid cells of 50 × 50 km^2^ (guided by spatial resolution of MERRA-2 dataset) to facilitate detailed spatial analysis of the extent of water reservoirs. Satellite images, accessed through Google Earth Engine, were filtered to include only those with less than 20% cloud cover. Mapping of the water bodies was based on the Normalized Difference Water Index (NDWI), a spectral index that differentiates water from land and vegetation by analyzing the reflectance differences between the green and near-infrared bands^50,55,56^. To mitigate the impact of outliers (such as floods, inundated terrains, and frequently irrigated lands), a median filter was applied to the annual data and a threshold value of 0.2 was adopted for the NDWI to provide a more accurate representation of annual water availability in water reservoirs.

Given the 10 m spatial resolution of Sentinel images, the smallest detectable water body in our analysis has a nominal area of 100 m^2^. However, to enhance the accuracy of our detection algorithm, we conservatively defined a water reservoir as an area consisting of at least three contiguous water pixels, thereby identifying water surfaces ranging from 300 to 100,000 m^2^ in agricultural areas of the MENA region. We implemented an object-based method in Google Earth Engine to delineate water features based on distinctive properties within the imagery, selectively filtering for potential small reservoirs in the defined size range. The extent of agricultural lands (excluding wetlands and large water bodies) were extracted from the ESA WorldCover v200 land cover map^57^. The MERIT Hydro dataset^58^ was further invoked to mask water canals and the reaches of ephemeral or intermittent streams in agricultural areas. Despite these detailed filtering processes designed to detect agricultural reservoirs, we acknowledge that a small fraction of multi-purpose structures (e.g., flood-control check dams that may also support irrigation) could remain in the dataset.

Details of reservoirs’ area extracted from Sentinel images enable us to estimate storage capacity of water reservoirs based on a power-law relation proposed by Mady et al.^10^. This relationship aligns well with the power-law estimates derived from area-volume power functions across 17 different global studies. We quantified the size distribution of reservoirs using 11 size classes. The first class includes reservoirs between 300 and 1,000 m^2^, while each subsequent class spans 10,000 m^2^. This classification allows for a more accurate representation of reservoir volume, particularly for small reservoirs (Fig. 1). Volume of reservoirs in each size class including *n* reservoirs is then estimated as *V* = 0.38×*n×A*^1.173^, where *A* ($\:=\sqrt{{a}_{min}\times\:{a}_{max}}$) is the representative area of reservoirs in each size class with minimum and maximum area of *a*_*min*_ and *a*_*max*_, respectively.

### Evaporative loss from water reservoirs

The evaporation model proposed by Aminzadeh et al.^30^ was employed to quantify evaporative fluxes from water reservoirs. The model solves the energy equation in depth of a water body accounting for absorption of radiative flux in depth of the reservoir (see Aminzadeh et al.^11,30^ for details):

1 $$\:\frac{\partial\:{T}_{w}}{\partial\:t}=\frac{\partial\:}{\partial\:z}\left(\left({\alpha\:}_{T,w}+{D}_{w}\right)\frac{\partial\:{T}_{w}}{\partial\:z}\right)+\frac{Q(z,t)}{{\rho\:}_{w}{c}_{w}}$$

where *T*_*w*_ is water temperature at time *t* and depth *z*, *α*_*T, w*_ and *D*_*w*_ are molecular and eddy thermal diffusivity, respectively, *Q* accounts for the absorption of radiative flux in depth of the water body, *ρ*_*w*_ is water density, and *c*_*w*_ is specific heat of water. Solution of Eq. (1) subjected to the radiation, sensible, and latent heat fluxes at the surface and quantification of water surface temperature (*T*_*ws*_) enable to calculate evaporation from the reservoir through the inherent coupling of water temperature and evaporative flux (temperature dependency of saturated vapor pressure)^59^:

2 $$\:E=86.4\times\:{10}^{6}\:\frac{0.622\:{\kappa\:}^{2}\:U}{{\rho\:}_{w}\:{R}_{d}\:{T}_{a}\:{\left[\text{l}\text{n}\left(\frac{z}{{z}_{0}}\right)\right]}^{2}}\left({e}_{s}\left({T}_{ws}\right)-{e}_{a}\right)$$

where *E* is evaporation rate, *U* is the wind speed, $\:\kappa\:$ is von Karman’s constant, *e*_*s*_ is saturated vapor pressure at the surface of water body, *e*_*a*_ is the vapor pressure within the air, *R*_*d*_ is the gas constant for dry air, *T*_*a*_ is the air temperature, *z* is the measurement height for air temperature and wind speed, and *z*_*0*_ is the roughness length. Model performance for estimating evaporative losses from water reservoirs was primarily evaluated using available evaporation data from water reservoirs in Spain and Iran. Details of the model evaluation are provided in the Supplementary Information (Figure S3).

Solving Eqs. (1) and (2) with the local atmospheric forcing parameters enables the calculation of evaporation losses from small agricultural reservoirs in the MENA region. Meteorological variables including wind speed, air temperature, humidity, and radiation, required for evaporation calculations were obtained from the Modern-Era Retrospective analysis for Research and Applications, Version 2 (MERRA-2) reanalysis datasets^31,32^ with resolution of 0.625° × 0.5°. MERRA-2 data were regridded using bilinear interpolation.

### Land cover, precipitation, and potential evapotranspiration data

The correlation analyses in Fig. 3 require estimates of the extent of cropland area and variation of aridity index (the ratio of precipitation to potential evapotranspiration) in the MENA region. Annual extent of cropland area was obtained from Esri’s global 10-m land-cover data^60^ providing the high resolution land cover information from 2017 to 2023. Due to the lack of land cover information in 2016 from this highly resolved inventory, the extent of cropland in 2016 was assumed similar to 2017. We extracted annual precipitation and potential evapotranspiration data from ERA5-Land Daily Aggregated dataset at spatial resolution of 0.1° ^29^, which was then upscaled by area-averaging native pixels within each 50 × 50 km^2^ grid cell to calculate the aridity index.

### Irrigation and livestock water use data

The agricultural water use in Fig. 4 was estimated based on annual irrigation and livestock water use. To quantify irrigation in the MENA region, we relied on the estimates of global irrigation water use proposed by Zhang et al.^54^. This dataset combines different satellite-derived soil moisture and precipitation products to develop a physical framework for estimating global irrigation water use. Livestock water use was estimated using the GLW 4 Gridded Livestock Density dataset which maps the global distribution of buffalo, cattle, sheep, goats, pigs, and chickens at a 10 km spatial resolution^40^. This dataset was upscaled and combined with species-specific annual water consumption^61^ to calculate livestock water use in 50 × 50 km^2^ grid cells.

## Supplementary Information

Below is the link to the electronic supplementary material.

Supplementary Material 1

## Data Availability

Sentinel-2 images were accessed through Google Earth Engine. Annual precipitation and potential evapotranspiration data were extracted from ERA5-Land Daily Aggregated dataset (10.24381/cds.e9c9c792). Land cover maps were accessed through ESA WorldCover v200 land cover map (10.5281/zenodo.7254221), and ESRI’s global 10-m land-cover data (10.1109/IGARSS47720.2021.9553499). The MERIT Hydro dataset is available through Google Earth Engine. Global moisture trajectory datasets can be accessed via 10.1594/PANGAEA.912710. Global irrigation water use was extracted from 10.11888/hydro.tpdc.271220. Livestock water use was estimated using the GLW 4 Gridded Livestock Density dataset (https://data.apps.fao.org/catalog/dataset/9d1e149b-d63f-4213-978b-317a8eb42d02). MERRA-2 reanalysis datasets are available at https://gmao.gsfc.nasa.gov/reanalysis/MERRA-2/data_access/.

## Code availability

The Google Earth Engine script for analyzing Sentinel-2 images, and all the necessary codes for reproducing the main results are available via GitHub at https://github.com/ghi-tuhh/water-storage-paradox-mena.
