# Supplementary material for: Water storage paradox of reservoir expansion and evaporative losses in the MENA region
Source: Sci Rep. 2025 Oct 1;15:34297. doi: 10.1038/s41598-025-21859-w (PMC12489012; doi:10.1038/s41598-025-21859-w)
Supplement: Supplementary file 1 — Supplementary Material 1 [file 41598_2025_21859_MOESM1_ESM.docx]

*Supplementary Information*

**Water Storage Paradox of Reservoir Expansion and Evaporative Losses in the MENA Region**

Milad Aminzadeh^1,2*^, Sankeerth Narayanaswamy^1,2^, Hannes Nevermann^1,2^, Matteo Zampieri^3,4^, Ibrahim Hoteit^3^, Paolo D'Odorico^5^, Amir AghaKouchak^6,7^, Kaveh Madani^7^, Nima Shokri^1,2*^

^1^Institute of Geo-Hydroinformatics, Hamburg University of Technology, Hamburg, Germany

^2^United Nations University Hub on Engineering to Face Climate Change at the Hamburg University of Technology, United Nations University Institute for Water, Environment and Health (UNU-INWEH), Hamburg, Germany

^3^Physical Sciences and Engineering Division, King Abdullah University of Science and Technology, Thuwal, Saudi Arabia

^4^Climate Change Center (CCC), National Center for Meteorology (NCM), Jeddah, Saudi Arabia

^5^Department of Environmental Science, Policy, and Management, University of California, Berkeley, CA, USA

^6^Department of Civil and Environmental Engineering, University of California, Irvine, CA, USA

^7^United Nations University Institute for Water, Environment and Health (UNU-INWEH), Richmond Hill, Ontario, Canada

***Corresponding authors:**

Milad Aminzadeh, email: [milad.aminzadeh@tuhh.de](mailto:milad.aminzadeh@tuhh.de)

Nima Shokri, email: [nima.shokri@tuhh.de](mailto:nima.shokri@tuhh.de)

Institute of Geo-Hydroinformatics

Hamburg University of Technology

Am Schwarzenberg-Campus 3 (E)

21073 Hamburg, Germany

**Supplementary Information:**

- The extent of small agricultural reservoirs and their storage capacity
- Model evaluation for estimating evaporation from water reservoirs
- Evaporation from agricultural reservoirs
- Fate of evaporated water from small reservoirs
- Correlations between the extent of water reservoirs and climatic factors

**The extent of small agricultural reservoirs and their storage capacity**

Figures S1 and S2 illustrate the distribution of small water reservoirs and the variation in their storage capacity across 50×50 km^2^ grid cells in agricultural regions of MENA during the study period from 2016 to 2023.


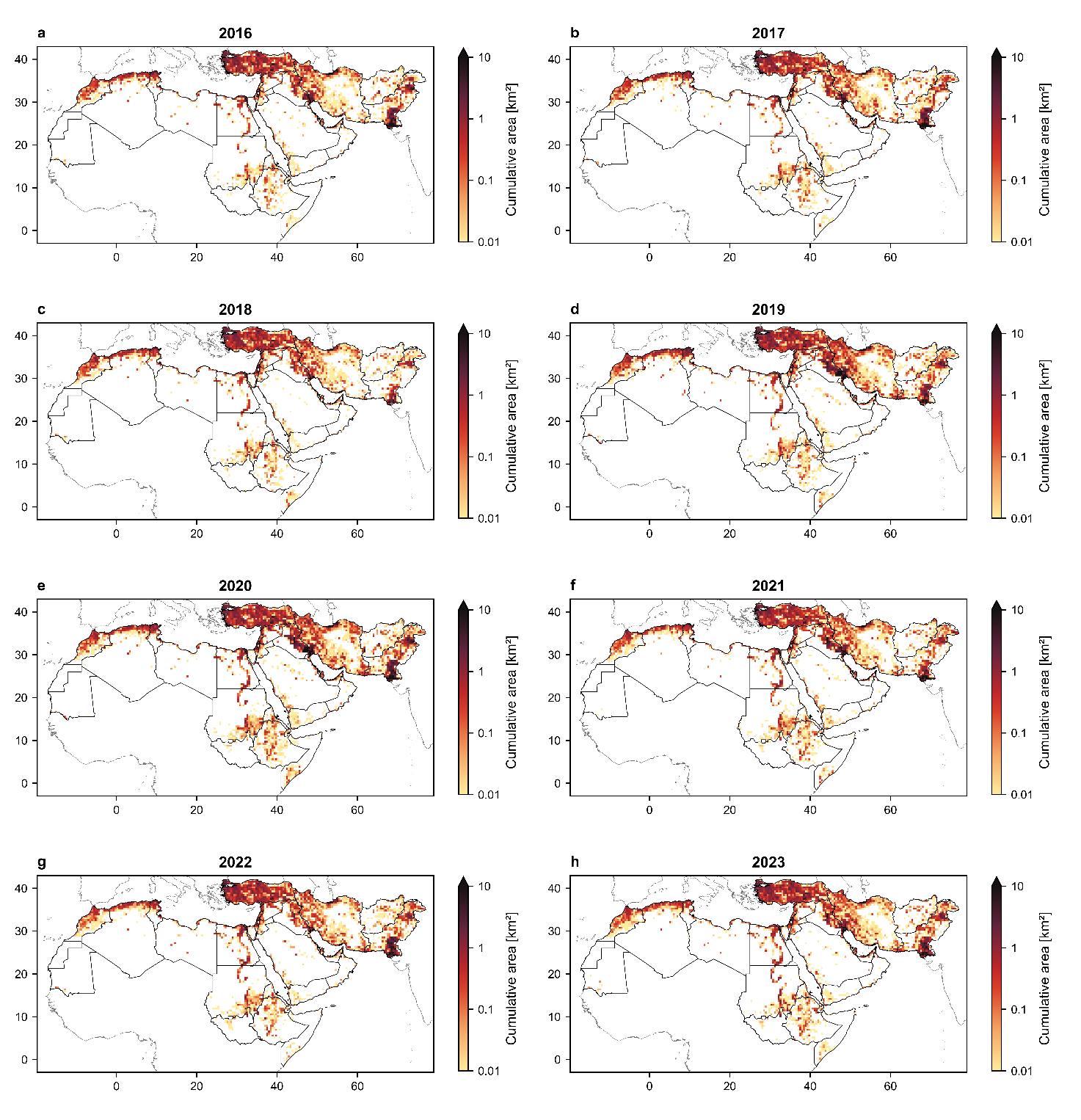


**Figure S1.** Spatial distribution of small water reservoirs (300-100,000 m^2^) in agricultural areas of the MENA region extracted from Sentinel-2 satellite images from 2016 to 2023. The logarithmic color bar indicates the cumulative area of reservoirs in each 50×50 km^2^ grid cell. Lowest and highest total area of the reservoirs in MENA were detected in 2018 and 2020, respectively.


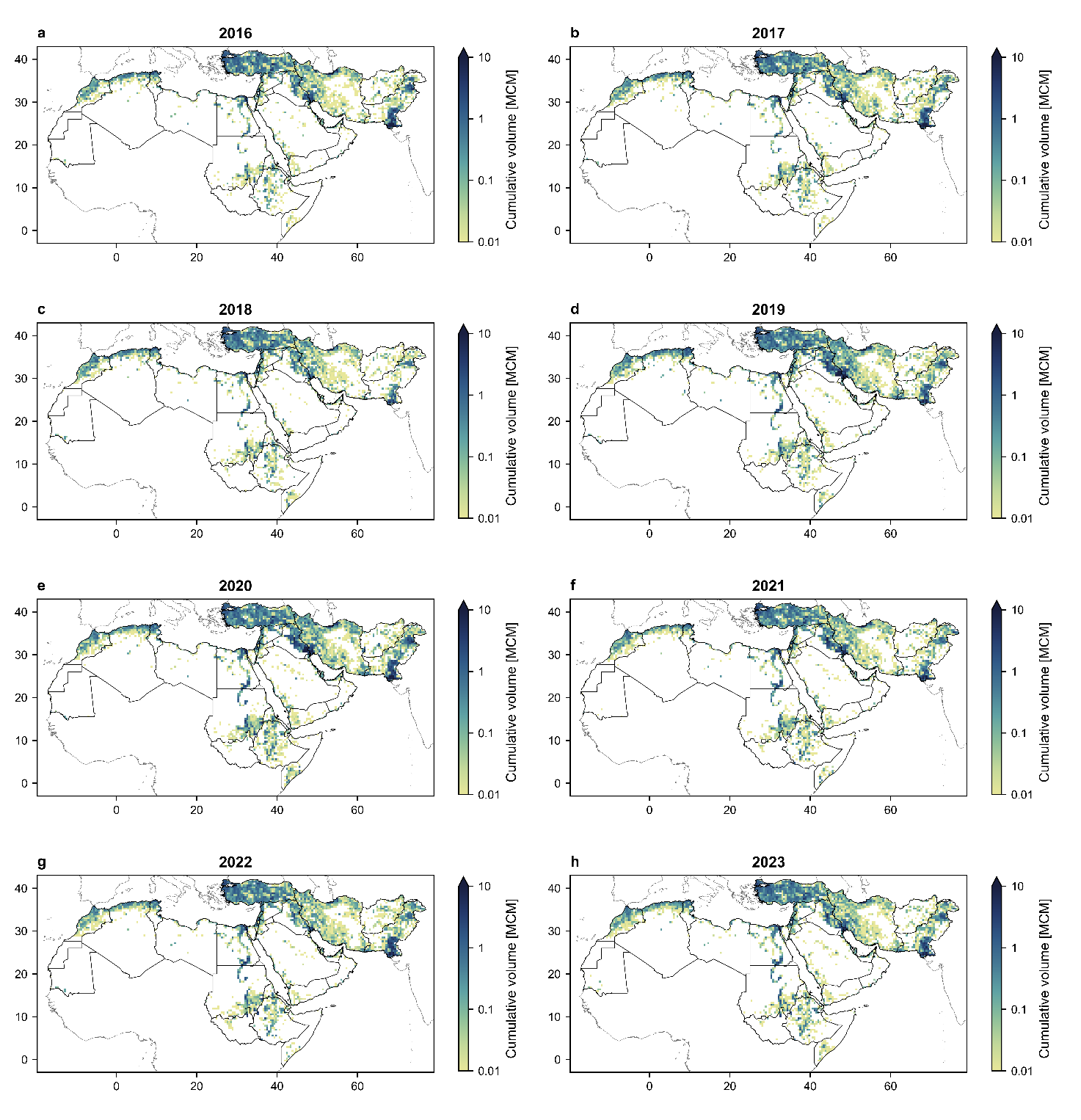


**Figure S2.** Variation of the cumulative storage of the reservoirs in agricultural areas of the MENA region during the study period from 2016 to 2023. The logarithmic color bar indicates the cumulative volume of reservoirs in each 50×50 km^2^ grid cell in million cubic meters (MCM). The volume of individual reservoirs was estimated based on reservoir surface area, using a power-law relationship proposed by Mady et al.^1^.

**Model evaluation for estimating evaporation from water reservoirs**

Model performance for estimating evaporation from small agricultural reservoirs was primarily assessed using measured data from water reservoirs in Spain and Iran. The first reservoir was in Cartagena, southeastern Spain (37°41′ N, 0°57′ W), with a surface area of 2,400 m^2^ and a depth of 5 m ^1^. Water level changes due to evaporation were monitored with a pressure transducer from April 2007 to March 2008. Due to the absence of detailed on-site meteorological data, wind speed, solar radiation, air temperature, and humidity were obtained from the MERRA-2 reanalysis dataset for the modeling process. The second reservoir, located in a dry and warm region of central Iran (32°42′ N, 51°31′ E), had a surface area of 25 m^2^ and a depth of 2 m ^2^. Evaporation was monitored using a pressure transducer, supported by detailed meteorological data collected from an on-site compact weather station between April 2019 and March 2020. Both reservoirs were lined with waterproof materials on the bottom and side walls. Figure S3 shows the comparison between the measured and modeled values of monthly evaporation from the reservoirs.


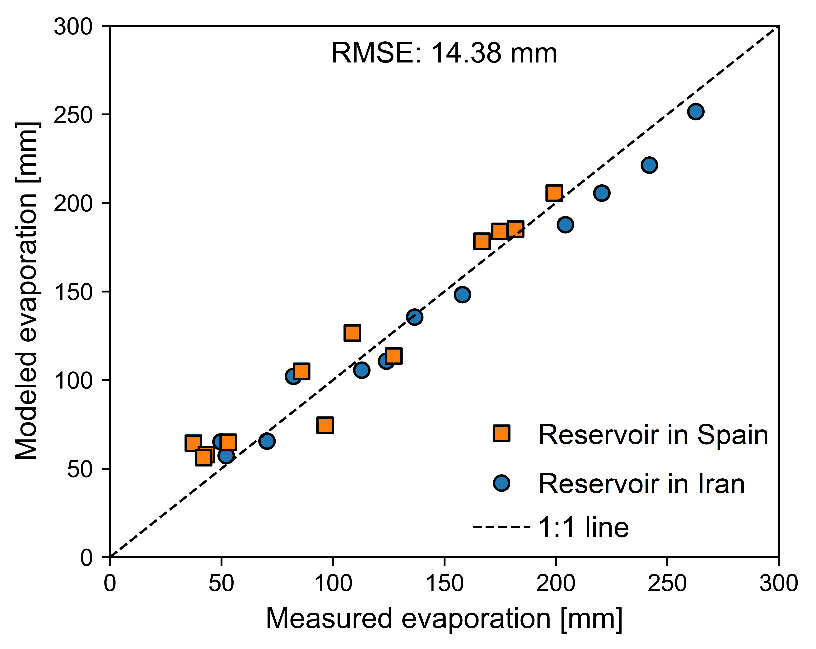


**Figure S3.** Comparison between modeled and measured monthly evaporation from the reservoirs in Spain and Iran.

**Evaporation from agricultural reservoirs**

Annual evaporation rate and cumulative evaporative loss from small reservoirs in each 50×50 km^2^ grid cell of agricultural regions (2016 - 2023) are shown in Figures S4 and S5, respectively. Figure S6 investigates the impact of reservoir depth on annual evaporation loss from reservoirs in MENA.


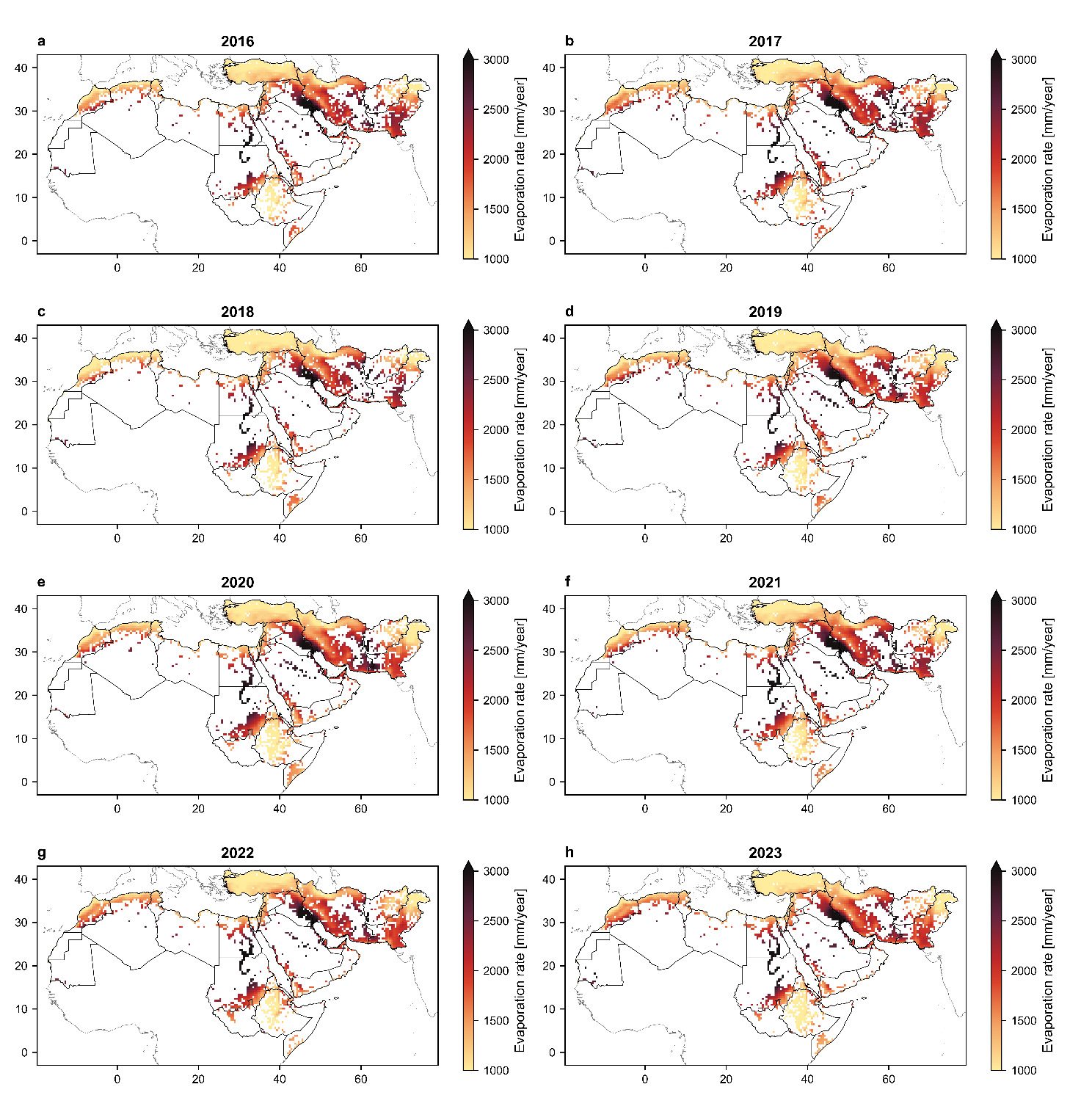


**Figure S4.** Spatial variation in evaporation rates from small agricultural reservoirs in the MENA region from 2016 to 2023. The results present modeled annual evaporation rates for a typical water reservoir with a depth of 3 m in each 50×50 km^2^ grid cell subjected to local atmospheric conditions (wind, radiation, air temperature, and humidity).


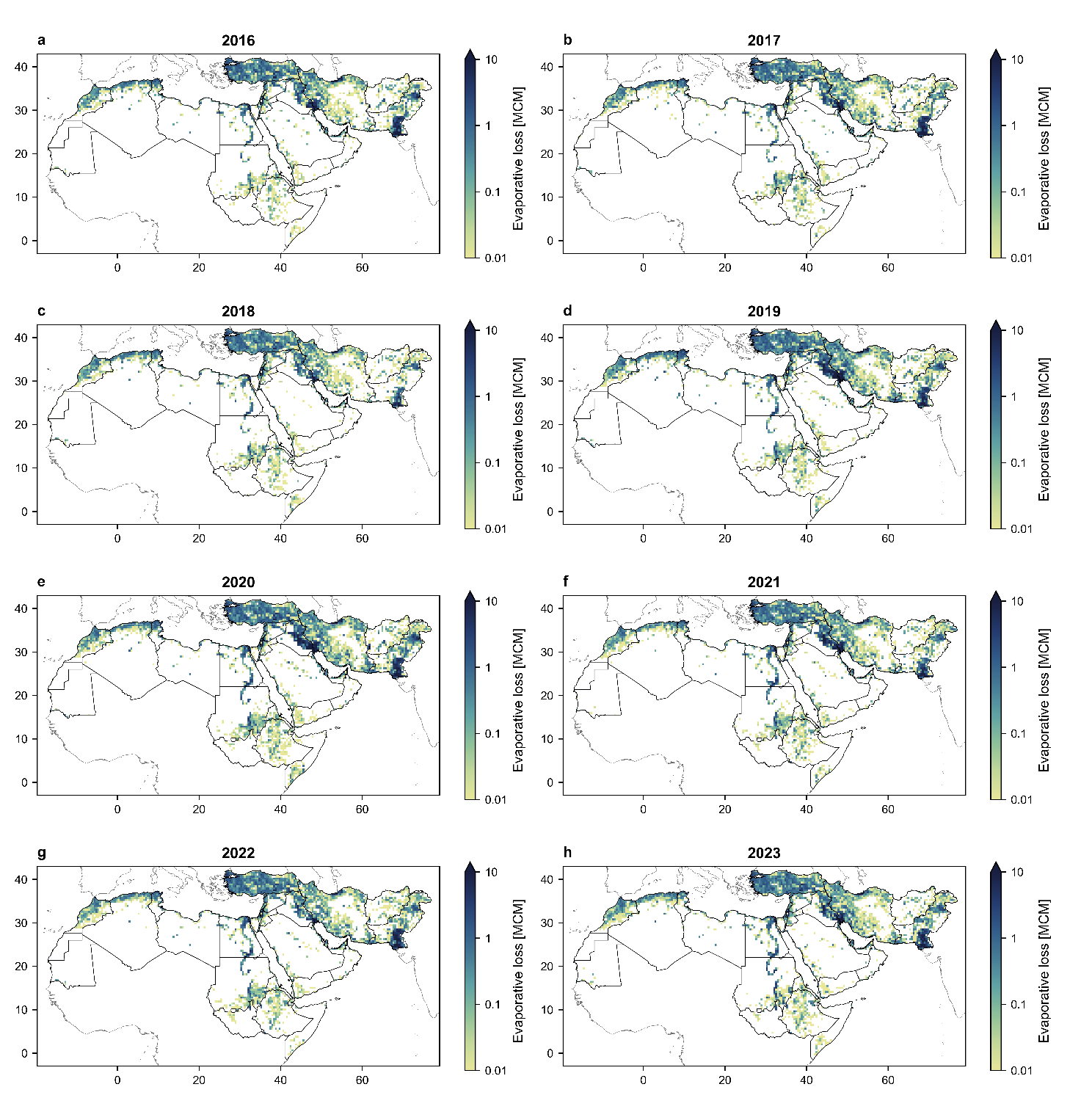


**Figure S5.** Changes in cumulative evaporative losses from small reservoirs in the MENA region from 2016 to 2023. The results were derived by multiplying the cumulative surface area of reservoirs within each 50×50 km^2^ grid cell (Figure S1) by the evaporation rate calculated for that cell (Figure S4).


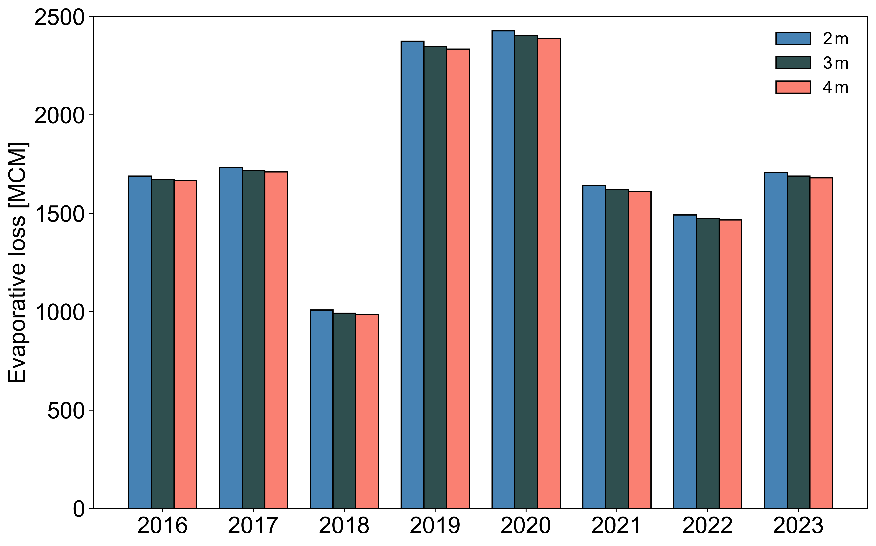


**Figure S6.** The impact of reservoir depth on modeled annual evaporation losses from small water reservoirs in the MENA region from 2016 to 2023. The findings suggest a slight increase in total evaporative losses as the depth of the typical water reservoir for modeling evaporation rate within each 50×50 km^2^ grid cell decreases.

**Fate of evaporated water from small reservoirs**

The fate of evaporated water can be determined through atmospheric numerical models or by analyzing datasets of atmospheric moisture trajectories^3^. We used the trajectories dataset computed by Tuinenburg et al.^4^ (hereafter T2020). This dataset was generated through a Lagrangian moisture-tracking model that utilized hourly wind speed and direction data across 25 vertical layers of the atmosphere. This information was sourced from the ERA5 reanalysis^5^ and spans the period from 2008 to 2017, when most of the reservoirs were already established. The reanalysis data results from a procedure called data assimilation which blends atmospheric circulation model equations and observations to create a self-consistent dataset as close as possible with the observed variables, even if they are sparse and discontinuous^5^. Therefore, the moisture trajectories derived from ERA5 can be considered our best proxy of reality.

The T2020 dataset has been utilized in numerous studies to measure local moisture recycling^6^, examine the non-local effects of vegetation changes on the water cycle^7–9^, explore the potential of forest management in drought mitigation^10^, define transboundary atmospheric watersheds to improve governance^11^, evaluate both the local and transboundary impacts of current and potential future irrigation in the Middle East^12^, and identify moisture recycling patterns of evaporation from reservoirs in the transboundary Helmand Basin, shared by Afghanistan and Iran^13^ (similar to the present study).

The trajectories dataset partially covers the study period. However, atmospheric circulation associated to the moisture trajectories in the MENA region displayed negligible variation over time when considering decadal means^12^. Therefore, we feel justified to use the T2020 dataset to assess the average fate of evaporation from the reservoirs in the MENA region over the study period. Nevertheless, we acknowledge the uncertainties associated with the methodology when interpreting the results. The overall effect of the reservoirs’ evaporation is then obtained by superimposing the precipitation footprint of each reservoir at the monthly level.

Figure S7 delineates the results of our analysis indicating the spatial distribution of annual precipitation that originates from the evaporated water from the small reservoirs in MENA. We found that most of the water is transported eastward, following the mean atmospheric flow in the middle latitude. This water precipitates over the main orographic features that it finds along its path, namely the Taurus-Zagros mountain chains and the Himalaya. Part of the water is also routed south, following the northern branch of the Hadley circulation and contributing to the tropical rain band over the Asir and Sarat mountains in the Southwest edge of the Arabian Peninsula and over the Ethiopian Highlands. Overall, the main beneficiaries of the evaporated water from small reservoirs in the MENA region are India (15.4%), followed by Iran (8.2%), Türkiye (6.4%), Ethiopia (5.8%) and Russia (5.0%). During the summer, the transfer of moisture (originating from small reservoirs in MENA) to India becomes particularly significant (24.2%) comparing to other countries.

It is important to note that these estimates may be affected by methodological uncertainties and correspond to relatively low precipitation rates. Nevertheless, the primary objective of the analysis was to trace the fate of evaporated water from small reservoirs in MENA, revealing that most of it is transported to distant regions far from its source. This finding offers valuable insights for regional water budgeting.


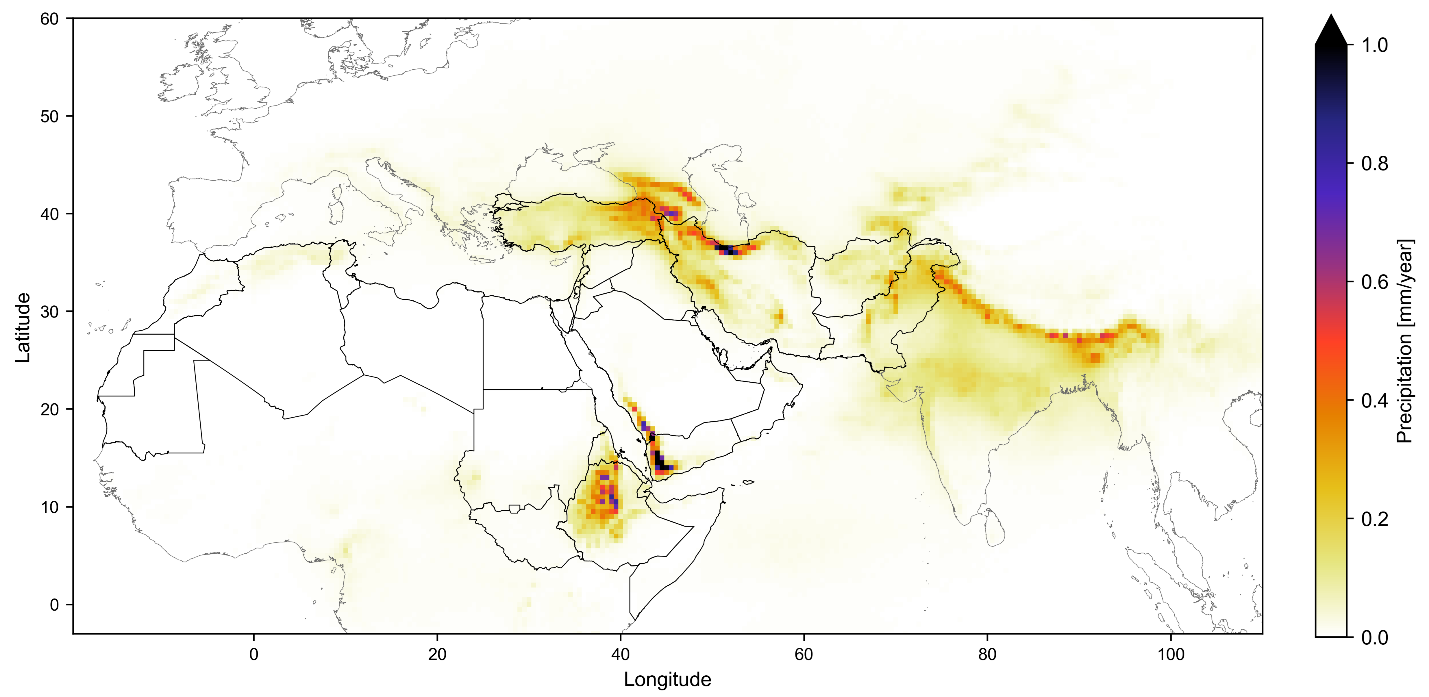


**Figure S7.** Annual precipitation resulting from the atmospheric moisture recycling of the evaporation from the small agricultural reservoirs in the MENA region.

**Correlations between the extent of water reservoirs and climatic factors**

To further investigate the climatic drivers of reservoir expansion, we performed a partial correlation analysis between annual reservoir area and climatic variables (precipitation and temperature). Results (Figure S8) show that reservoir extent is positively correlated with precipitation, while exhibiting a negative correlation with temperature. These results indicate that reservoir expansion in the study region is primarily governed by water availability (i.e., precipitation), whereas higher temperatures tend to suppress reservoir extent through enhanced evaporative demand.


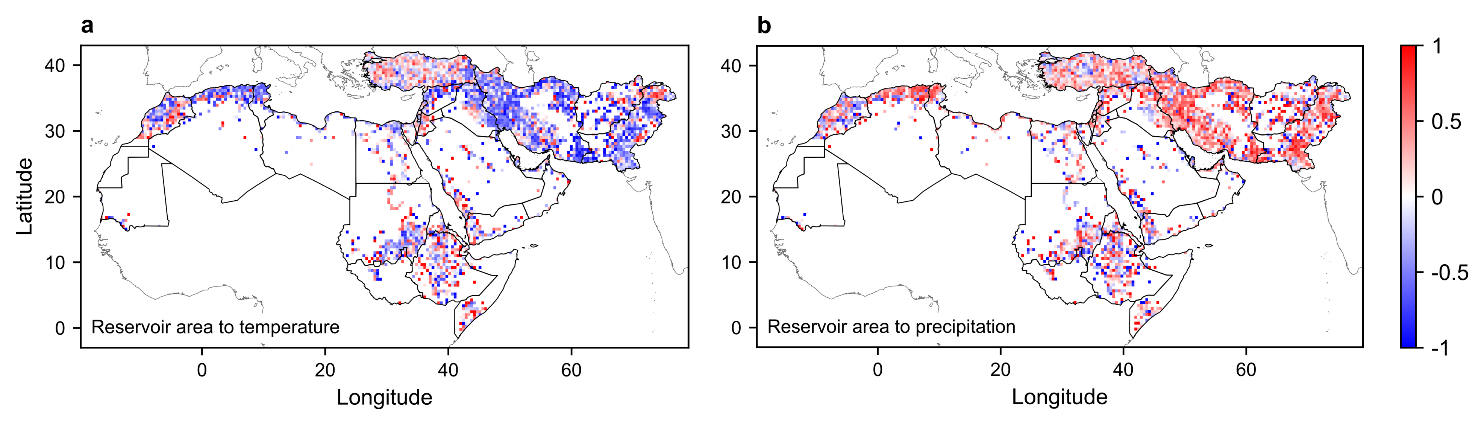


**Figure S8.** Spatial variation of Pearson correlation coefficient between the cumulative surface area of water reservoirs and temperature (a), and precipitation (b).

**References**

1. Gallego-Elvira, B., Baille, A., Martin-Gorriz, B., Maestre-Valero, J. F. & Martínez-Alvarez, V. Energy balance and evaporation loss of an irrigation reservoir equipped with a suspended cover in a semiarid climate (south-eastern Spain). *Hydrological Processes* **25**, 1694–1703 (2011).

2. Bakhtiar, M., Aminzadeh, M., Taheriyoun, M., Or, D. & Mashayekh, E. Effects of floating covers used for evaporation suppression on reservoir physical, chemical and biological water quality parameters. *Ecohydrology* **15**, e2470 (2022).

3. van der Ent, R. J., Tuinenburg, O. A., Knoche, H.-R., Kunstmann, H. & Savenije, H. H. G. Should we use a simple or complex model for moisture recycling and atmospheric moisture tracking? *Hydrology and Earth System Sciences* **17**, 4869–4884 (2013).

4. Tuinenburg, O. A., Theeuwen, J. J. E. & Staal, A. High-resolution global atmospheric moisture connections from evaporation to precipitation. *Earth System Science Data* **12**, 3177–3188 (2020).

5. Hersbach, H. *et al.* The ERA5 global reanalysis. *Quarterly Journal of the Royal Meteorological Society* **146**, 1999–2049 (2020).

6. Theeuwen, J. J. E., Staal, A., Tuinenburg, O. A., Hamelers, B. V. M. & Dekker, S. C. Local moisture recycling across the globe. *Hydrology and Earth System Sciences* **27**, 1457–1476 (2023).

7. Baudena, M., Tuinenburg, O. A., Ferdinand, P. A. & Staal, A. Effects of land-use change in the Amazon on precipitation are likely underestimated. *Global Change Biology* **27**, 5580–5587 (2021).

8. Cui, J. *et al.* Global water availability boosted by vegetation-driven changes in atmospheric moisture transport. *Nat. Geosci.* **15**, 982–988 (2022).

9. Hoek van Dijke, A. J. *et al.* Shifts in regional water availability due to global tree restoration. *Nat. Geosci.* **15**, 363–368 (2022).

10. Tuinenburg, O. A., Bosmans, J. H. C. & Staal, A. The global potential of forest restoration for drought mitigation. *Environ. Res. Lett.* **17**, 034045 (2022).

11. Rockström, J., Mazzucato, M., Andersen, L. S., Fahrländer, S. F. & Gerten, D. Why we need a new economics of water as a common good. *Nature* **615**, 794–797 (2023).

12. Zampieri, M. *et al.* Leveraging atmospheric moisture recycling in Saudi Arabia and neighboring countries for irrigation and afforestation planning. *Reg Environ Change* **24**, 124 (2024).

13. Nevermann, H., Madani, K., Zampieri, M., Hoteit, I. & Shokri, N. Struggling over water, losing it through evaporation: The case of Afghanistan and Iran. *Journal of Environmental Management* **375**, 124319 (2025).
